# Single-Cell Transcriptomic Analysis Reveals Cell Heterogeneity and Altered Signaling Pathways in Jellyfish Sting Patients

**DOI:** 10.3390/md23090358

**Published:** 2025-09-15

**Authors:** Zhen Qin, Zhengfeng Hao, Chun Wang, Ning Lu, Peiju Qiu, Su Wang, Rilei Yu

**Affiliations:** 1 Key Laboratory of Marine Drugs, Ministry of Education of China, School of Medicine and Pharmacy, Ocean University of China, Qingdao 266003, China; 2 PLA Naval Medical Center, Naval Medical University (Second Military Medical University), Shanghai 200433, China; 3 School of Pharmacy, Bengbu Medical University, Bengbu 233030, China; 4 Center for Translational Neuromedicine, University of Rochester Medical Center, Rochester, NY 14642, USA

**Keywords:** jellyfish stings, scRNA-seq, cell heterogeneity, CD74+ monocytes, MMP9+ monocytes, S100A12+ neutrophils

## Abstract

Jellyfish stings induce a range of symptoms, from localized irritation to life-threatening systemic reactions, yet the underlying immune mechanisms remain poorly understood. This study employed single-cell RNA sequencing (scRNA-seq) to analyze peripheral blood mononuclear cells (PBMCs) from a severely affected patient and healthy controls, uncovering the immune landscape at single-cell resolution and identifying the signaling pathways. We identified 11 major immune cell types, with a marked increase in CD14+ monocytes (81.86% of total cells) and significant reductions in T cells, B cells, and CD16+ monocytes in the envenomated patient. Subclustering revealed six monocyte and four neutrophil subsets, each displaying distinct functional profiles. Patient monocytes were enriched for MMP9+ and RETN+ subsets, associated with leukocyte migration and inflammation, whereas healthy controls exhibited CD74+ monocytes linked to oxidative phosphorylation. Neutrophils in the patient were predominantly LTF+ and S100A12+, implicating inflammatory and immune regulatory pathways. These findings provide a detailed single-cell atlas of immune dysregulation post-jellyfish sting, highlighting the pivotal roles of MMP9+ monocytes and S100A12+ neutrophils in driving inflammation. This study offers potential therapeutic targets for mitigating severe immune responses in jellyfish envenomation.

## 1. Introduction

Jellyfish, belonging to the phylum Cnidaria, are widely distributed marine organisms known for their ecological abundance and taxonomic diversity. Characterized by a bell-shaped body adorned with tentacles and oral arms, they harbor specialized stinging cells called nematocysts, which deliver a complex cocktail of bioactive toxins upon contact with predators or prey. The venom composition is highly heterogeneous, comprising proteins, peptides, enzymes, tetramine, potent anesthetics, histamines, serotonin, and other components [1,2]. These toxins exhibit a broad spectrum of biological activities, such as hemolytic, cardiotoxic, neurotoxic, dermal-muscular toxicities, as well as enzymatic and antioxidant properties [3]. Envenomation in humans can lead to a range of clinical manifestations—from localized pain, erythema, and blistering to systemic complications such as cardiovascular instability, neurotoxicity, acute kidney injury, and, in severe cases, multi-organ failure and death [4,5].

Despite the increasing incidence of jellyfish stings worldwide, the molecular and immunological mechanisms underlying their pathophysiology remain poorly understood. This knowledge gap has significantly impeded the development of targeted and effective therapeutic interventions. A deeper understanding of the host immune response to cnidarian toxins is therefore essential for identifying key cellular and molecular drivers of disease progression. Recent advances in single-cell transcriptomics have opened new avenues for dissecting complex immune responses with unprecedented resolution. Single-cell RNA sequencing (scRNA-seq) enables the profiling of gene expression at the individual cell level, revealing cellular heterogeneity, rare cell populations, and dynamic transcriptional states that are often masked in bulk RNA sequencing analyses [6,7,8,9,10,11,12,13,14].

In the context of envenomation, traditional bulk transcriptomic approaches provide only an averaged view of gene expression across heterogeneous cell populations, obscuring critical cell-type-specific responses. In contrast, scRNA-seq offers a powerful platform to uncover the precise immune cell subsets and signaling pathways perturbed by jellyfish toxins. Previous studies have demonstrated that jellyfish venom components trigger robust inflammatory responses, including the upregulation of pro-inflammatory cytokines and chemokines, which promote leukocyte recruitment and activation [15,16,17,18,19]. Moreover, systemic manifestations such as acute myocardial injury, pulmonary edema, and arrhythmias suggest a profound dysregulation of immune and vascular homeostasis following envenomation [20]. However, the specific immune cell types and intercellular communication networks involved in these processes remain largely uncharacterized.

Recent applications of scRNA-seq in immunology have revolutionized our understanding of host responses in infectious, autoimmune, and inflammatory diseases, enabling the discovery of novel cell states, regulatory circuits, and therapeutic targets [14,21,22,23,24,25,26,27,28]. In the context of toxin exposure, this technology holds particular promise for identifying distinct immune subpopulations that drive inflammation or tissue damage. For instance, scRNA-seq can reveal differentially expressed marker genes, reconstruct signaling pathways, and infer cell–cell interactions that underlie pathological immune activation [6,29,30,31,32,33,34,35,36,37,38].

In this study, we applied scRNA-seq to peripheral blood mononuclear cells (PBMCs) from a patient with severe jellyfish envenomation and matched healthy controls to comprehensively characterize the immune response at single-cell resolution. We aimed to (i) define alterations in immune cell composition, with a focus on monocytes, neutrophils, and lymphocytes; (ii) identify differentially expressed genes and their functional enrichment in inflammatory and migratory pathways; and (iii) infer cell–cell communication networks that may contribute to systemic immune dysregulation.

Our findings reveal a profound shift in immune homeostasis following jellyfish stings, dominated by the expansion of pro-inflammatory MMP9+ and RETN+ monocyte subsets and the activation of S100A12+ neutrophils—cell populations strongly associated with tissue damage and inflammation. This work not only advances our understanding of the pathophysiology of jellyfish envenomation but also highlights potential molecular targets for therapeutic intervention. By combining marine toxin biology with cutting-edge immunogenomics, this study illustrates how marine-derived challenges can contribute to the discovery of novel immune mechanisms and, ultimately, the development of bioinspired therapeutics.

## 2. Results

### 2.1. Overview of Single-Cell Transcriptomic Profiling of PBMCs

To investigate the immune landscape following severe jellyfish envenomation, we performed single-cell RNA sequencing (scRNA-seq) on peripheral blood mononuclear cells (PBMCs) from one patient with systemic symptoms and three healthy individuals. A total of four PBMC samples were collected, processed via density gradient centrifugation, and subjected to high-throughput scRNA-seq. This approach enabled high-resolution profiling of gene expression at the single-cell level, allowing for the identification of cell-type-specific transcriptional signatures.

**Figure 1:**
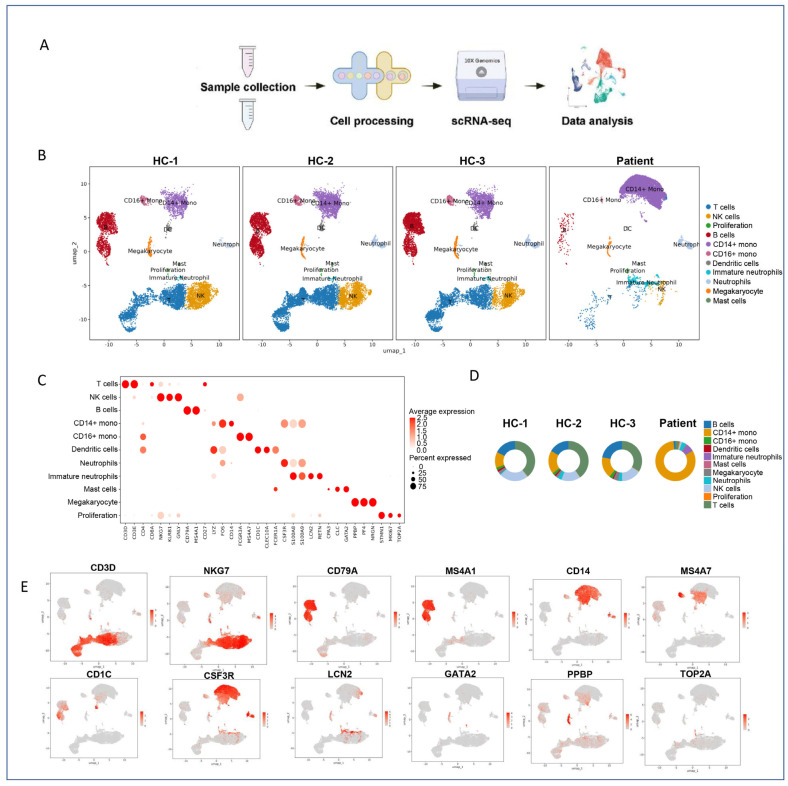
Overview and cell type analysis of PBMC single-cell transcriptomics. (**A**) Schematic representation of the study design and workflow. The figure outlines the steps from patient sample processing to single-cell RNA sequencing and downstream analysis. (**B**) UMAP projection of sampled cell types. A UMAP plot illustrating the dimensionality reduction of different cell types identified within the PBMC samples. Each dot represents a single cell, colored by its annotated cell type to visualize the distribution and clustering of cellular diversity. (**C**) Dot plot of canonical marker genes. A dot plot displaying the expression of classical marker genes across various cell types. The size of each dot indicates the percentage of cells expressing the gene, while the color intensity indicates the average expression level. (**D**) Pie charts illustrating the proportion of 11 major cell types across the four samples. Each bar represents a sample, and the colors correspond to different cell types. Pie charts showing the relative proportions of eleven major cell types identified in the PBMCs of the four individual samples. (**E**) Feature plots for selected canonical marker genes providing a spatial representation of gene expression levels on the UMAP plot. These plots help to identify the specific cell types associated with the expression of these marker genes.

Bioinformatic analyses included quality control, read alignment, count matrix generation, normalization, and dimensionality reduction. Unsupervised clustering was applied to delineate distinct cell populations within the integrated dataset. Based on canonical marker gene expression, we identified 11 major immune cell types: T cells, B cells, natural killer (NK) cells, CD14+ monocytes, CD16+ monocytes, dendritic cells (DCs), neutrophils, immature neutrophils, megakaryocytes, mast cells, and proliferating cells (Figure 1A,B). The clustering and annotation workflow is summarized in Figure 1A, and the Uniform Manifold Approximation and Projection (UMAP) visualization revealed well-separated populations, reflecting the cellular heterogeneity present in human PBMCs.

To validate the accuracy of cell type annotations, we generated a dot plot showing the average expression and detection frequency of established lineage-specific markers. This analysis confirmed robust expression of CD3E in T cells, CD19 in B cells, CD14 in classical monocytes, FCGR3A (CD16) in non-classical monocytes and neutrophils, KLRD1 (CD94) in NK cells, and S100A8/A9 in myeloid populations (Figure 1C). These results support the reliability of our cell type classification and provide a solid foundation for downstream analyses.

### 2.2. Altered Immune Cell Composition in Jellyfish Envenomation

Comparative analysis of cell type proportions revealed dramatic shifts in the immune landscape of the jellyfish sting patient compared to healthy controls. In the control group, T cells were the predominant population (37.68% of total cells), followed by B cells (18.80%), NK cells (17.80%), and CD14+ monocytes (16.58%), with neutrophils representing only a minor fraction (2.62%)—consistent with typical PBMC composition.

In striking contrast, the patient exhibited a profound skewing toward myeloid lineage expansion. CD14+ monocytes became the dominant cell type, constituting 81.86% of all PBMCs—an over fivefold increase compared to controls. Immature neutrophils were the second-most abundant population (6.42%), suggesting active myelopoiesis or emergency granulopoiesis in response to envenomation. The proportions of T cells, B cells, and CD16+ monocytes were significantly reduced in the patient compared to the healthy controls (Figure 1D).

These findings demonstrate a systemic immune imbalance characterized by monocytic expansion and lymphopenia, indicative of a strong innate immune activation and potential immunosuppressive state in the adaptive compartment. This shift mirrors patterns observed in severe inflammatory or sepsis-like conditions, highlighting the intensity of the host response to jellyfish toxins.

### 2.3. Feature Plot of Canonical Marker Genes

To further validate the identity and spatial distribution of annotated cell clusters, we visualized the expression of key lineage-defining genes on the UMAP embedding. Feature plots confirmed the specific and restricted expression of classical markers within their expected clusters. These plots confirmed the localized expression of marker genes within their respective cell clusters, further validating the cell type annotations (Figure 1E).

### 2.4. Subclustering and Functional Annotation of Monocytes

**Figure 2:**
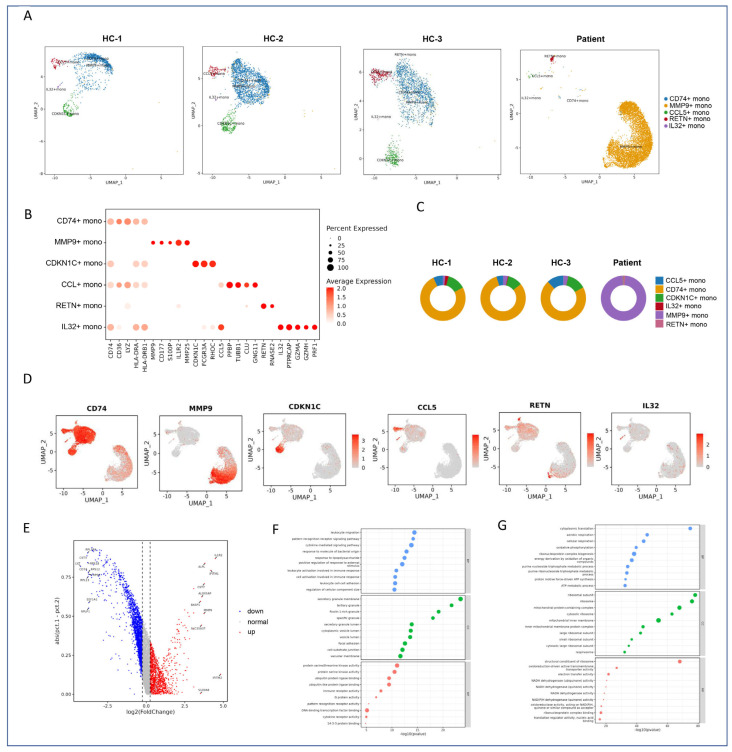
Subclustering and functional analysis of monocytes. (**A**) UMAP plot depicting the distribution of monocyte (Mono) subtypes across the four samples. Each color represents a distinct monocyte subtype. (**B**) Dot plot showing the expression of marker genes for each monocyte subtype. The dot size and color intensity represent the percentage of cells expressing the gene and the average expression level, respectively. (**C**) Pie charts display the proportion of six monocyte subtypes across the four samples. Each bar represents a sample, and the colors correspond to different monocyte subtypes. (**D**) Feature plot illustrating the spatial distribution of marker genes for each monocyte subtype on the UMAP plot. UMAP_1 and UMAP_2 represent the two primary dimensions resulting from dimensionality reduction. (**E**) Volcano plot highlighting differentially expressed genes between MMP9+ monocytes and CD74+ monocytes. Significantly upregulated genes are marked in red. (**F**) Pathway enrichment analysis of upregulated genes in MMP9+ monocytes. The bar plot shows the top enriched pathways. (**G**) Pathway enrichment analysis of upregulated genes in CD74+ monocytes. The bar plot shows the top enriched pathways.

To further dissect the heterogeneity within monocyte populations, we performed subclustering analysis using UMAP, which revealed six distinct monocyte subtypes (Figure 2A). These subtypes included MMP9+ monocytes and CD74+ monocytes, among others, each showing clear spatial separation in the UMAP embedding, indicating significant transcriptional diversity within the monocyte lineage.

#### 2.4.1. Subtype-Specific Gene Signatures of Monocytes

To characterize the gene expression profiles specific to each subtype, a dot plot analysis was conducted (Figure 2B). MMP9+ monocytes uniquely expressed MMP9, while CD74+ monocytes showed elevated levels of CD74. Additionally, other subtypes such as CDKN1C+ monocytes, CCL5+ monocytes, RETN+ monocytes, and IL32+ monocytes exhibited characteristic marker genes indicative of their functional roles. Quantitative assessment of the proportions of these subtypes across samples demonstrated variability, with MMP9+ monocytes being particularly enriched in the patient sample (Figure 2C). Feature plots further validated the distinct spatial localization of these marker genes within the UMAP coordinates, confirming the accuracy of our annotations. Conversely, CD74+ monocytes were more prevalent in healthy controls, with upregulation of genes like LYZ and CD74, which are involved in oxidative phosphorylation and aerobic respiration (Figure 2D).

#### 2.4.2. Differential Gene Expression Analysis

To explore the functional differences between MMP9+ and CD74+ monocytes, differential gene expression analysis was performed and visualized using a volcano plot (Figure 2E). MMP9+ monocytes exhibited significant upregulation of inflammatory response genes, including IL1R2 and IFITM1, suggesting their involvement in acute inflammation. In contrast, CD74+ monocytes were enriched in genes related to antigen processing and presentation pathways, indicative of their role in immune surveillance and regulation.

Gene set enrichment analysis (GSEA) corroborated these findings, revealing that MMP9+ monocytes were significantly enriched in cytokine signaling and immune response pathways (Figure 2F), whereas CD74+ monocytes displayed activation of oxidative phosphorylation and aerobic respiration pathways (Figure 2G). These results highlight the distinct functional roles of these monocyte subtypes in orchestrating immune responses.

#### 2.4.3. Classification of Monocyte Subclusters

The six identified monocyte subclusters were classified based on their gene expression profiles and functional annotations.

CD74+ monocytes (Clusters 0, 7): Dominant in healthy controls, associated with oxidative phosphorylation and aerobic respiration, reflecting their role in maintaining cellular homeostasis. MMP9+ monocytes (Clusters 1, 2): Enriched in patients, linked to leukocyte migration (MMP9, IL1R2) and inflammatory responses, highlighting their involvement in acute immune reactions. CDKN1C+ monocytes (Cluster 3): Marked by cell cycle regulatory genes, suggesting a role in proliferative processes or tissue repair. CCL5+ monocytes (Cluster 4): Featured chemokine-related gene expression, indicating their involvement in chemotactic recruitment of immune cells. RETN+ monocytes (Cluster 5): Promoted myeloid/neutrophil chemotaxis, likely contributing to the mobilization of neutrophils during inflammation. IL32+ monocytes (Cluster 6): Enriched in interleukin-associated cytotoxicity pathways, potentially playing a role in enhancing immune-mediated cytotoxic effects.

### 2.5. Subclustering and Functional Annotation of Neutrophils

**Figure 3:**
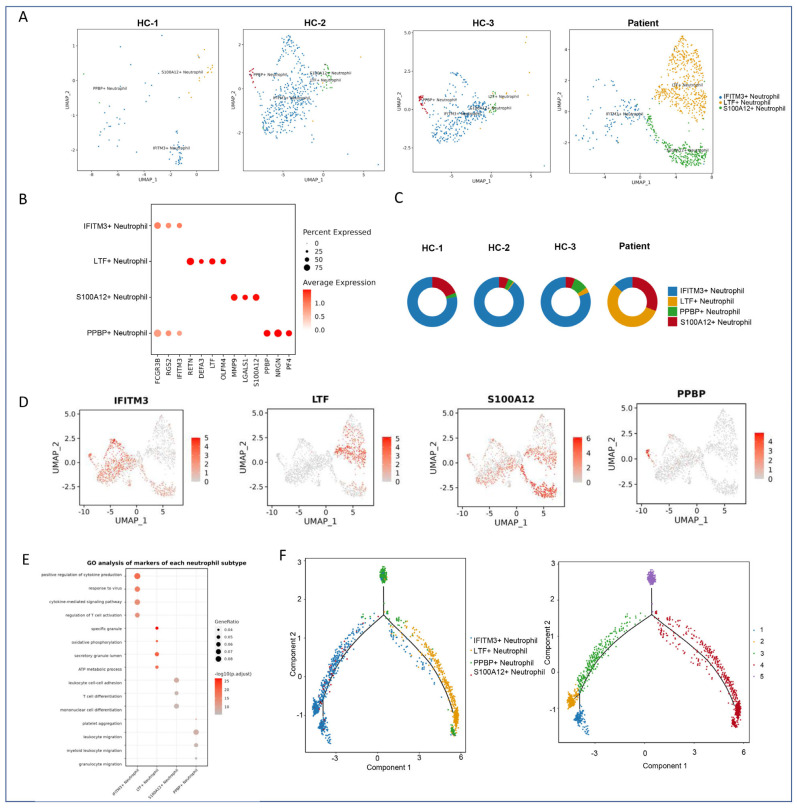
Subclustering and functional analysis of neutrophils. (**A**) UMAP plot showing the distribution of neutrophil subtypes across the four samples. Each color represents a distinct neutrophil subtype. (**B**) Dot plot displaying the expression of marker genes for each neutrophil subtype. The dot size and color intensity represent the percentage of cells expressing the gene and the average expression level, respectively. (**C**) Pie charts illustrate the proportion of four neutrophil subtypes across the four samples. Each bar represents a sample, and the colors correspond to different neutrophil subtypes. (**D**) Feature plot showing the spatial distribution of marker genes for each neutrophil subtype on the UMAP plot. UMAP_1 and UMAP_2 represent the two primary dimensions resulting from dimensionality reduction. (**E**) Dot plot summarizing the functional pathways associated with each neutrophil subtype. The dot size represents the significance of the pathway, and the color indicates the enrichment score. (**F**) Monocle pseudotime analysis of neutrophil subtypes. A trajectory analysis using Monocle to infer the potential differentiation paths or states of neutrophil subtypes over pseudotime. Component 1 and Component 2 in the pseudotime analysis reflect the dominant trajectory components.

To investigate the heterogeneity within neutrophil populations, we performed subclustering analysis using UMAP, which identified four distinct neutrophil subtypes (Figure 3A). These subtypes included IFITM3+, LTF+, S100A12+, and PPBP+ neutrophils, each exhibiting unique transcriptional signatures.

#### 2.5.1. Subtype-Specific Gene Signatures of Neutrophils

Dot plot analysis was conducted to identify subtype-specific markers (Figure 3B). The pie chart highlights the inter-sample variability in subtype abundance, demonstrating significant differences between the jellyfish sting patient and healthy controls. Differential expression analysis revealed that MMP9+ monocytes were significantly enriched in the jellyfish sting patient (Figure 3C). Feature plots further confirmed the spatial segregation of these marker genes within the UMAP space (Figure 3D).

#### 2.5.2. Functional Pathway Enrichment Analysis

Pathway enrichment analysis highlighted several key signaling pathways activated or suppressed in response to jellyfish toxins. Notably, pathways associated with leukocyte migration, cytokine signaling, and inflammatory responses were upregulated in the patient’s immune cells. Additionally, there was evidence of suppression in pathways related to metabolic processes and oxidative phosphorylation, suggesting a shift in cellular energy metabolism following envenomation (Figure 3E). These findings underscore the systemic impact of jellyfish toxins on immune cell function and metabolism.

Specifically, IFITM3+ neutrophils (Cluster 0) are enriched in antiviral defense and leukocyte migration. LTF+ neutrophils (Cluster 1) are associated with granule formation and antiviral gene expression, indicating an active role in innate immunity. S100A12+ neutrophils (Cluster 2) are implicated in leukocyte adhesion, T-cell proliferation, and myeloid differentiation, suggesting a critical role in inflammatory amplification. PPBP+ neutrophils (Cluster 3) are linked to platelet activation and granulocyte motility, contributing to vascular repair and immune cell trafficking.

#### 2.5.3. Pseudotime Trajectory Analysis

Pseudotime trajectory analysis (Monocle) suggested a differentiation continuum from immature to mature neutrophil states (Figure 3F). In the patient sample, neutrophils were predominantly LTF+ and S100A12+, with S100A12+ subsets driving leukocyte adhesion and inflammatory responses. Additionally, our exploration of the temporal dynamics and differentiation trajectories of neutrophil subpopulations suggested that LTF+ neutrophils might represent an immature state, which was primarily found in the patient. S100A12+ neutrophils appeared to be an intermediate state, while IFITM3+ neutrophils were considered mature. This differentiation trajectory implies that jellyfish toxins may disrupt normal neutrophil maturation, leading to an accumulation of immature cells in the patient.

### 2.6. Altered Cell–Cell Communication Networks

To investigate the changes in cell–cell communication networks following jellyfish envenomation, we performed comprehensive ligand–receptor interaction analysis. This analysis revealed significant alterations in key signaling pathways, particularly those involving chemokines and cytokines, within the monocytes and neutrophils of the patient.

#### 2.6.1. Heatmap Visualization of Interaction Strength and Specificity

**Figure 4:**
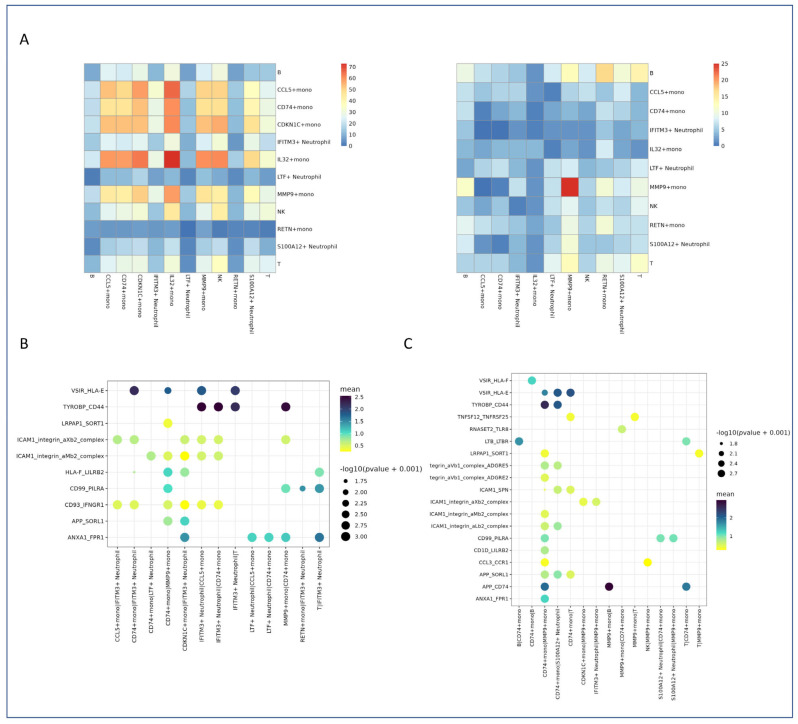
Cell–cell communication analysis in PBMC samples. (**A**) Heatmap showing the interaction strength between cell types in healthy controls (HCs, **left**) and the patient (**right**). The color intensity represents the interaction strength. (**B**) Dot plot summarizing the key interaction pairs in healthy controls (HCs). The dot size represents the interaction strength, and the color indicates the significance. (**C**) Dot plot summarizing the key interaction pairs in the patient. The dot size represents the interaction strength, and the color indicates the significance.

We performed cell–cell communication analysis to identify potential interactions between different cell types. A heatmap was generated to visualize the strength and specificity of interactions in healthy control (HC) and patient samples. Notable differences in interaction patterns are observed between the HC and patient groups, suggesting altered cell communication in disease conditions. Specifically, MMP9+ monocytes and S100A12+ neutrophils showed increased interactions, potentially contributing to enhanced inflammatory responses and impaired immune function. These findings highlight the importance of considering cell–cell interactions when investigating the pathophysiology of jellyfish stings. Additionally, several interactions were upregulated or downregulated in the patient, suggesting a shift in immune regulatory mechanisms (Figure 4A).

#### 2.6.2. Dot Plot Analysis of Key Interaction Pairs in Healthy Controls

To further characterize these interactions, we generated a dot plot to highlight key ligand–receptor pairs that are highly active in healthy controls (Figure 4B). This analysis identified specific interactions predominantly involved in immune regulation and homeostasis, which underscores the importance of maintaining immune homeostasis in healthy individuals.

#### 2.6.3. Dot Plot Analysis of Key Interaction Pairs in Patient Samples

Similarly, a dot plot was generated to highlight key interaction pairs in the patient sample (Figure 4C). This analysis revealed significant alterations in cell–cell communication in patient samples, with certain interactions being upregulated or downregulated compared to healthy controls.

These findings indicate that altered cell–cell communication may play a crucial role in the pathogenesis of systemic inflammation following jellyfish envenomation.

## 3. Discussion

Our study presents the first comprehensive single-cell transcriptomic atlas of the human immune response following severe jellyfish envenomation. By leveraging single-cell RNA sequencing (scRNA-seq), we achieved unprecedented resolution in characterizing immune cell heterogeneity, uncovering profound alterations in cellular composition, functional states, and intercellular communication networks in a patient with systemic toxicity. While bulk RNA sequencing has been instrumental in profiling immune responses in envenomation and inflammatory diseases, it inherently masks cellular diversity and transitional states [39]. In contrast, scRNA-seq enables the dissection of rare subsets, differentiation trajectories, and cell-type-specific signaling pathways [40], making it uniquely suited to unravel the complexity of venom-induced immune dysregulation—a domain previously unexplored in human marine envenomation.

Although scRNA-seq has advanced our understanding of immune dynamics in conditions such as sepsis [41], its application to toxin-mediated injuries remains limited. Our work bridges this critical gap by systematically analyzing PBMCs from a severe jellyfish sting patient and three healthy controls, revealing key immunological perturbations that underlie the pathophysiology of envenomation.

### 3.1. Immune Cell Composition Remodeling and Pro-Inflammatory Monocyte Expansion

One of the most striking findings was a dramatic shift in immune cell composition: CD14+ monocytes expanded to constitute 81.86% of total PBMCs in the patient, while T cells, B cells, and classical neutrophil populations were markedly reduced. This profound reprogramming suggests that jellyfish toxins drive a systemic skewing of hematopoiesis or peripheral mobilization toward myeloid lineage dominance, consistent with emergency myelopoiesis observed in severe infection or trauma [42,43,44,45,46,47].

The expansion of MMP9+ monocytes—a subset characterized by high expression of IL1R2, IFITM1, and MMP9—points to their central role in amplifying inflammation and tissue remodeling. MMP9 encodes a matrix metalloproteinase capable of degrading extracellular matrix components, facilitating leukocyte infiltration into injured tissues [48]. Its co-expression with IL1R2, a decoy receptor that modulates IL-1 signaling, suggests a dual regulatory mechanism, promoting early inflammatory recruitment while potentially dampening excessive IL-1-driven pathology. This finding resolves longstanding uncertainty about the cellular sources of proteolytic activity in envenomation [49,50], implicating monocytes—not just neutrophils—as key mediators of tissue damage.

In contrast, healthy controls exhibited a dominance of CD74+ monocytes, which is enriched in genes involved in oxidative phosphorylation and antigen processing (LYZ, CD74, HLA class II), reflecting a homeostatic, immunoregulatory phenotype. The replacement of this population by pro-inflammatory MMP9+ and RETN+ monocytes in the patient underscores a fundamental shift from immune surveillance to inflammatory activation.

### 3.2. Neutrophil Subsets and Impaired Maturation Trajectory

Subclustering revealed distinct neutrophil subpopulations with divergent functional profiles. Healthy individuals showed higher proportions of IFITM3+ and PPBP+ neutrophils, associated with antiviral defense, granulocyte motility, and vascular repair. Conversely, the patient exhibited a striking accumulation of LTF+ and S100A12+ neutrophils.

Notably, pseudotime analysis suggests that LTF+ neutrophils represent an immature state, S100A12+ cells an intermediate stage, and IFITM3+ cells a mature, terminally differentiated phenotype. The predominance of immature and intermediate subsets in the patient implies that jellyfish toxins may inhibit neutrophil maturation, possibly through direct cytotoxic effects or disruption of differentiation signals in the bone marrow or circulation [51,52]. Immature neutrophils are known to exhibit reduced phagocytic capacity and microbial killing efficiency [53,54], which may contribute to secondary infection risk and prolonged inflammation.

Moreover, S100A12+ neutrophils—known to activate TLR4/NF-κB signaling via RAGE binding [55,56]—were not only expanded but also functionally primed for leukocyte adhesion, myeloid proliferation, and cytokine production. Their enrichment suggests a role in inflammatory amplification and immune cell crosstalk, further destabilizing immune homeostasis.

### 3.3. Altered Cell–Cell Communication and Inflammatory Synergy

Cell communication analysis revealed a rewired interaction network in the patient. Most notably, enhanced ligand–receptor interactions between MMP9+ monocytes and S100A12+ neutrophils suggest a synergistic pro-inflammatory circuit. These cells co-express molecules involved in leukocyte migration (CCL3/4–CCR1/5), cytokine signaling (IL1–IL1R2), and extracellular matrix remodeling (MMP9–TIMP2), forming a positive feedback loop that sustains inflammation.

For example, MMP9 facilitates tissue penetration by degrading basement membranes, while S100A12 acts as a chemoattractant and adhesion promoter, collectively enhancing immune cell recruitment and activation. This crosstalk likely contributes to the systemic inflammatory response syndrome (SIRS)-like phenotype observed in severe stings.

Additionally, interactions between neutrophils and dendritic cells were significantly upregulated in the patient, potentially promoting aberrant antigen presentation and adaptive immune activation. Such dysregulated crosstalk may underlie the persistent inflammation and delayed recovery seen in some envenomation cases.

### 3.4. Therapeutic Implications and Future Directions

Our findings highlight several promising therapeutic targets. First, MMP9 inhibition could mitigate tissue damage and leukocyte infiltration, with existing inhibitors (e.g., doxycycline, marimastat) offering potential for repurposing [57,58]. Second, targeting the IL-1 pathway, particularly via blockade of IL-1R2 signaling or S100A12–RAGE interaction, may help break the inflammatory cycle [59,60]. Third, interventions that promote neutrophil maturation—such as G-CSF or modulation of key transcription factors (e.g., C/EBPε, PU.1)—could restore immune competence and accelerate recovery [61,62].

Furthermore, disrupting the MMP9+ monocyte–S100A12+ neutrophil axis may represent a novel strategy to dampen inflammation without broad immunosuppression. Validating these targets in larger cohorts and in vivo models will be essential for translation.

### 3.5. Limitations and Outlook

While this study provides deep mechanistic insights, it is based on a single patient. Future work should expand to multi-patient cohorts across different envenomation severities and time points to capture dynamic immune trajectories. Additionally, integrating proteomic and functional assays will be crucial to validate transcriptomic findings and assess cellular behavior ex vivo.

Nonetheless, our data establish a foundational framework for understanding how marine toxins reprogram the immune system at single-cell resolution. The identification of specific pathogenic subsets and their communication networks opens new avenues for precision immunomodulation in envenomation and other toxin-mediated inflammatory conditions.

## 4. Materials and Methods

### 4.1. Human Sample Collection

Blood was obtained from a jellyfish-sting-induced CF patient and three healthy human donors. The stings were caused by a Sand Sea Jellyfish (*Rhopilema esculentum*). Multiple lower limb regions (thighs and calves) were exposed. The incident occurred while swimming in coastal waters. The study was conducted in accordance with the Declaration of Helsinki and approved by the Institutional Ethics Committees of the PLA Naval Medical Center (Clinical Trial No. AF-HEC-054) and Ocean University of China (OUC-HM-2025-042). Written informed consent was obtained from all participants prior to sample collection.

### 4.2. Isolation of PBMCs and Preparation of Single-Cell Suspensions

PBMCs were isolated by density gradient centrifugation using Ficoll-Paque Plus medium (GE Healthcare) (Cytiva, Marlborough, MA, USA) and washed with Ca/Mg-free PBS. To remove the red blood cells, 2 mL red blood cell lysis buffer was added at 25 °C for 10 min. The solution was then centrifuged at 500× *g* for 5 min and suspended in PBS. The blood samples were centrifuged at 400× *g* for 5 min at 4 °C, and the supernatant was discarded. After the red blood cells had been removed, PBMCs were isolated by centrifugation at 400× *g* for 10 min at 4 °C. The supernatant was discarded and the PBMCs were resuspended by phosphate-buffered saline to obtain a single-cell suspension. Cells were processed immediately for 10× Genomics single-cell capture and cDNA synthesis. Libraries were sequenced on an Illumina NovaSeq 6000 platform following quality control assessment.

### 4.3. Processing scRNA-Seq Data

Single-cell suspensions were processed using the 10× Genomics Chromium platform. Libraries were prepared according to the manufacturer’s protocol and sequenced on an Illumina NovaSeq 6000 platform. Raw sequencing data were aligned to the human reference genome using Cell Ranger (v7.0.1) to obtain the Unique Molecular Identifier (UMI) matrix, which was further imported into R (v4.2.2) and processed with the Seurat package (v4.3.2). Cells with a detected gene number of <200 or >5000 or a high mitochondrial transcript ratio (>10%) were excluded. The top 2000 highly variable genes were extracted to perform principal component analysis (PCA), and the top 30 principal components (PCs) were used for cluster analysis. After normalization and scaling, the batch effect between patient samples was then removed using harmony. The batch-corrected matrix was used for further analysis and visualization. Cell types were annotated by the SingleR package (v1.2.4) and then checked manually.

### 4.4. Identification of Marker Genes

To identify differentially expressed marker genes for each cell type, the FindAllMarkers function in Seurat was used under the default parameters. Marker genes were selected as those with adjusted *p* values less than 0.05, an average logFC larger than 0.25, and a percentage of cells with expression higher than 0.25. Gene Ontology (GO), Kyoto Encyclopedia of Genes and Genomes (KEGG), and GSEA were performed with the R package clusterProfiler v3.18.0, using all detected genes from the entire scRNA-seq library as background. Terms were enriched with the nominal *p* value < 0.05 and false discovery rate (*q* value) < 0.05.

### 4.5. Cell Type Annotation

The cell type identity of each cluster was determined according to the expression of canonical markers found in the DEGs using well-known cell-type-specific markers. Dot plots displaying the expression of markers used to identify each cell type were generated by Seurat v3.1.2 DotPlot.

### 4.6. Pathway Enrichment Analysis

To investigate the potential functions of DEGs, Gene Ontology (GO) and Kyoto Encyclopedia of Genes and Genomes (KEGG) analysis were used with the “clusterProfiler” R package 3.16.1. Pathways with a p_adj value of less than 0.05 were considered significantly enriched. Gene Ontology gene sets including molecular function (MF), biological process (BP), and cellular component (CC) categories were used as reference.

### 4.7. Quality Control, Dimension Reduction, and Clustering

Seurat v 3.1.2 was used for quality control, dimensionality reduction, and clustering. For each sample dataset, we filtered the expression matrix by the following criteria: (1) cells with gene counts of less than 200 or with a gene count in the top 2% were excluded; (2) cells with a UMI count in the top 2% were excluded; (3) cells with a mitochondrial content in the top 10% were excluded; (4) genes expressed in less than 5 cells were excluded. After filtering, 93,143 cells were retained for the downstream analyses, with on average 1524 genes and 2875 UMIs per cell. The gene expression matrix was normalized and scaled using the functions NormalizeData and ScaleData. The top 2000 variable genes were selected by FindVariableFeatures for PCA. Cells were separated into 18 clusters by FindClusters, using the top 20 principal components and a resolution parameter of 0.1. Cell clusters were visualized using UMAP with the Seurat functions RunTSNE and RunUMAP.

### 4.8. Differentially Expressed Gene (DEG) Analysis (Seurat)

To identify differentially expressed genes (DEGs), we used the Seurat FindMarkers function based on the Wilcoxon rank sum test with the default parameters and selected the genes expressed in more than 10% of the cells in both of the compared groups of cells and with an average log (Fold Change) value greater than 0.25, identifying these as DEGs. The adjusted *p* value was calculated using the Bonferroni correction and the value 0.05 was used as the criterion to evaluate the statistical significance.

### 4.9. UCell Gene Set Scoring

Gene set activity scores were calculated using the UCell R package (v1.1.0), a non-parametric, rank-based method robust to batch effects and library size variation. For each predefined gene signature, UCell computes a Mann–Whitney U statistic based on the relative expression ranks of the genes within each cell. This approach is particularly suitable for large-scale, multi-sample scRNA-seq datasets. Signature scores were used to infer activation states of biological pathways across cell types.

### 4.10. Cell–Cell Communication Analysis

We used cellphoneDB (v.4.0.0) to infer cell–cell interactions of selected ligand–receptor pairs between cells. The potential interaction strength between two cell subsets was predicted based on the expression of ligand–receptor pairs. The enriched ligand–receptor interactions between two cell subsets were calculated based on a permutation test. We extracted significant ligand–receptor pairs with a *p* value < 0.01. Key signaling circuits (e.g., inflammatory, chemotactic) were visualized using chord plots and hierarchical clustering.

## 5. Conclusions

In conclusion, our single-cell transcriptomic analysis provides a high-resolution portrait of the immune landscape in peripheral blood following jellyfish envenomation. We reveal substantial alterations in the abundance and activation states of monocytes and neutrophils, underscoring the potent immunomodulatory impact of jellyfish venom components. These findings extend the current understanding of venom-induced inflammatory responses and highlight key cellular pathways that may contribute to tissue injury and systemic symptoms. By identifying dysregulated immune mechanisms at single-cell resolution, this study not only advances the pathophysiological knowledge of jellyfish stings but also opens new avenues for the development of targeted therapeutics—potentially informing the design of anti-venom strategies or host-directed interventions. As marine toxins continue to serve as rich sources of bioactive molecules, our work reinforces the value of integrating single-cell technologies into marine drug and toxin research.

## Data Availability

The original contributions presented in this study are included in the article. Further inquiries can be directed to the corresponding authors.

## Abbreviations

The following abbreviations are used in this manuscript:

scRNA-seq	Single-cell RNA sequencing
PBMC	Peripheral blood mononuclear cells
CF	Cardiac function
UMAP	Uniform Manifold Approximation and Projection
NK	Natural killer
DC	Dendritic cells
GSEA	Gene set enrichment analysis
GO	Gene Ontology
KEGG	Kyoto Encyclopedia of Genes and Genomes
HC	Healthy control
S100A12	S100 calcium-binding protein A12
MMP9	Matrix metalloproteinase
DEGs	Differentially expressed genes

## References

[1] Naveed M., Chan M.W.H., Aslam S., Wang F., Sajjad A., Ullah A., Saleem N., Haider M.S., Arija V. (2024). Nutritional composition assessment and antimicrobial activity of Catostylus perezi, jellyfish blooms along the coast of Pakistan: An awareness to avoid food neophobia in Pakistan. Nat. Prod. Res..

[2] Killi N., Mariottini G.L. (2018). Cnidarian Jellyfish: Ecological Aspects, Nematocyst Isolation, and Treatment Methods of Sting. Results Probl. Cell Differ..

[3] Malacarne P.F., Menezes T.N., Martins C.W., Naumann G.B., Gomes H.L., Pires R.G.W., Figueiredo S.G., Campos F.V. (2018). Advances in the characterization of the Scorpaena plumieri cytolytic toxin (Sp-CTx). Toxicon.

[4] Baysoy A., Bai Z., Satija R., Fan R. (2023). The technological landscape and applications of single-cell multi-omics. Nat. Rev. Mol. Cell Biol..

[5] Ruwanpathirana P., Priyankara D. (2022). Clinical manifestations of wasp stings: A case report and a review of literature. Trop. Med. Health.

[6] Zhang M., Hu S., Min M., Ni Y., Lu Z., Sun X., Wu J., Liu B., Ying X., Liu Y. (2021). Dissecting transcriptional heterogeneity in primary gastric adenocarcinoma by single cell RNA sequencing. Gut.

[7] Wang C., Yu Q., Song T., Wang Z., Song L., Yang Y., Shao J., Li J., Ni Y., Chao N. (2022). The heterogeneous immune landscape between lung adenocarcinoma and squamous carcinoma revealed by single-cell RNA sequencing. Signal Transduct. Target. Ther..

[8] Peroni E., Randi M.L., Rosato A., Cagnin S. (2023). Acute myeloid leukemia: From NGS, through scRNA-seq, to CAR-T. dissect cancer heterogeneity and tailor the treatment. J. Exp. Clin. Cancer Res..

[9] Choi Y.H., Kim J.K. (2019). Dissecting Cellular Heterogeneity Using Single-Cell RNA Sequencing. Mol. Cells.

[10] Zhao J., Zhang S., Liu Y., He X., Qu M., Xu G., Wang H., Huang M., Pan J., Liu Z. (2020). Single-cell RNA sequencing reveals the heterogeneity of liver-resident immune cells in human. Cell Discov..

[11] Massalha H., Bahar Halpern K., Abu-Gazala S., Jana T., Massasa E.E., Moor A.E., Buchauer L., Rozenberg M., Pikarsky E., Amit I. (2020). A single cell atlas of the human liver tumor microenvironment. Mol. Syst. Biol..

[12] Yang P., Luan M., Li W., Niu M., He Q., Zhao Y., Chen J., Mao B., Mou K., Li P. (2023). Single-cell transcriptomics reveals peripheral immune responses in non-segmental vitiligo. Front. Immunol..

[13] Abplanalp W.T., John D., Cremer S., Assmus B., Dorsheimer L., Hoffmann J., Becker-Pergola G., Rieger M.A., Zeiher A.M., Vasa-Nicotera M. (2021). Single-cell RNA-sequencing reveals profound changes in circulating immune cells in patients with heart failure. Cardiovasc. Res..

[14] Kim D., Kobayashi T., Voisin B., Jo J.H., Sakamoto K., Jin S.P., Kelly M., Pasieka H.B., Naff J.L., Meyerle J.H. (2020). Targeted therapy guided by single-cell transcriptomic analysis in drug-induced hypersensitivity syndrome: A case report. Nat. Med..

[15] McKellar D.W., Walter L.D., Song L.T., Mantri M., Wang M.F.Z., De Vlaminck I., Cosgrove B.D. (2021). Large-scale integration of single-cell transcriptomic data captures transitional progenitor states in mouse skeletal muscle regeneration. Commun. Biol..

[16] Gotts J.E., Matthay M.A. (2016). Sepsis: Pathophysiology and clinical management. BMJ.

[17] Feio-Azevedo R., Boesch M., Radenkovic S., van Melkebeke L., Smets L., Wallays M., Boeckx B., Philips G., Prata de Oliveira J., Ghorbani M. (2025). Distinct immunometabolic signatures in circulating immune cells define disease outcome in acute-on-chronic liver failure. Hepatology.

[18] Kronsten V.T., Tranah T.H., Pariante C., Shawcross D.L. (2022). Gut-derived systemic inflammation as a driver of depression in chronic liver disease. J. Hepatol..

[19] Miyabe C., Miyabe Y., Nagai J., Miura N.N., Ohno N., Chun J., Tsuboi R., Ueda H., Miyasaka M., Miyasaka N. (2019). Abrogation of lysophosphatidic acid receptor 1 ameliorates murine vasculitis. Arthritis Res. Ther..

[20] Berling I., Isbister G. (2015). Marine envenomations. Aust. Fam. Physician.

[21] Galeano Niño J.L., Wu H., LaCourse K.D., Kempchinsky A.G., Baryiames A., Barber B., Futran N., Houlton J., Sather C., Sicinska E. (2022). Effect of the intratumoral microbiota on spatial and cellular heterogeneity in cancer. Nature.

[22] Wu S.Z., Al-Eryani G., Roden D.L., Junankar S., Harvey K., Andersson A., Thennavan A., Wang C., Torpy J.R., Bartonicek N. (2021). A single-cell and spatially resolved atlas of human breast cancers. Nat. Genet..

[23] Yan N., Xie W., Wang D., Fang Q., Guo J., Chen Y., Li X., Gong L., Wang J., Guo W. (2024). Single-cell transcriptomic analysis reveals tumor cell heterogeneity and immune microenvironment features of pituitary neuroendocrine tumors. Genome Med..

[24] Tegowski M., Flamand M.N., Meyer K.D. (2022). scDART-seq reveals distinct m6A signatures and mRNA methylation heterogeneity in single cells. Mol. Cell.

[25] Jin H., Li M., Jeong E., Castro-Martinez F., Zuker C.S. (2024). A body-brain circuit that regulates body inflammatory responses. Nature.

[26] Mulder K., Patel A.A., Kong W.T., Piot C., Halitzki E., Dunsmore G., Khalilnezhad S., Irac S.E., Dubuisson A., Chevrier M. (2021). Cross-tissue single-cell landscape of human monocytes and macrophages in health and disease. Immunity.

[27] Thakore P.I., Schnell A., Huang L., Zhao M., Hou Y., Christian E., Zaghouani S., Wang C., Singh V., Singaraju A. (2024). BACH2 regulates diversification of regulatory and proinflammatory chromatin states in TH17 cells. Nat. Immunol..

[28] Jin C., Jiang P., Zhang Z., Han Y., Wen X., Zheng L., Kuang W., Lian J., Yu G., Qian X. (2024). Single-cell RNA sequencing reveals the pro-inflammatory roles of liver-resident Th1-like cells in primary biliary cholangitis. Nat. Commun..

[29] Hoogstrate Y., Draaisma K., Ghisai S.A., van Hijfte L., Barin N., de Heer I., Coppieters W., van den Bosch T.P.P., Bolleboom A., Gao Z. (2023). Transcriptome analysis reveals tumor microenvironment changes in glioblastoma. Cancer Cell.

[30] Guo S., Liu X., Zhang J., Huang Z., Ye P., Shi J., Stalin A., Wu C., Lu S., Zhang F. (2023). Integrated analysis of single-cell RNA-seq and bulk RNA-seq unravels T cell-related prognostic risk model and tumor immune microenvironment modulation in triple-negative breast cancer. Comput. Biol. Med..

[31] Fan Y., Bian X., Meng X., Li L., Fu L., Zhang Y., Wang L., Zhang Y., Gao D., Guo X. (2024). Unveiling inflammatory and prehypertrophic cell populations as key contributors to knee cartilage degeneration in osteoarthritis using multi-omics data integration. Ann. Rheum. Dis..

[32] Wang M., Liu X., Chang G., Chen Y., An G., Yan L., Gao S., Xu Y., Cui Y., Dong J. (2018). Single-Cell RNA Sequencing Analysis Reveals Sequential Cell Fate Transition during Human Spermatogenesis. Cell Stem Cell.

[33] Honardoost M.A., Adinatha A., Schmidt F., Ranjan B., Ghaeidamini M., Arul Rayan N., Gek Liang Lim M., Joanito I., Xiao Xuan Lin Q., Rajagopalan D. (2024). Systematic immune cell dysregulation and molecular subtypes revealed by single-cell RNA-seq of subjects with type 1 diabetes. Genome Med..

[34] Chen J., Larsson L., Swarbrick A., Lundeberg J. (2024). Spatial landscapes of cancers: Insights and opportunities. Nat. Rev. Clin. Oncol..

[35] Kim H., Kim K.E., Madan E., Martin P., Gogna R., Rhee H.W., Won K.J. (2024). Unveiling contact-mediated cellular crosstalk. Trends Genet..

[36] Sun Y., Zhang Z., Qiao Q., Zou Y., Wang L., Wang T., Lou B., Li G., Xu M., Wang Y. (2024). Immunometabolic changes and potential biomarkers in CFS peripheral immune cells revealed by single-cell RNA sequencing. J. Transl. Med..

[37] Zhang D., Wen Q., Zhang R., Kou K., Lin M., Zhang S., Yang J., Shi H., Yang Y., Tan X. (2024). From Cell to Gene: Deciphering the Mechanism of Heart Failure With Single-Cell Sequencing. Adv. Sci..

[38] Yang W., Wang P., Xu S., Wang T., Luo M., Cai Y., Xu C., Xue G., Que J., Ding Q. (2024). Deciphering cell-cell communication at single-cell resolution for spatial transcriptomics with subgraph-based graph attention network. Nat. Commun..

[39] Papalexi E., Satija R. (2018). Single-cell RNA sequencing to explore immune cell heterogeneity. Nat. Rev. Immunol..

[40] Stuart T., Butler A., Hoffman P., Hafemeister C., Papalexi E., Mauck W.M., Hao Y., Stoeckius M., Smibert P., Satija R. (2019). Comprehensive Integration of Single-Cell Data. Cell.

[41] Reyes M., Filbin M.R., Bhattacharyya R.P., Billman K., Eisenhaure T., Hung D.T., Levy B.D., Baron R.M., Blainey P.C., Goldberg M.B. (2020). An immune-cell signature of bacterial sepsis. Nat. Med..

[42] Peet C., Ivetic A., Bromage D.I., Shah A.M. (2020). Cardiac monocytes and macrophages after myocardial infarction. Cardiovasc. Res..

[43] Simats A., Zhang S., Messerer D., Chong F., Beşkardeş S., Chivukula A.S., Cao J., Besson-Girard S., Montellano F.A., Morbach C. (2024). Innate immune memory after brain injury drives inflammatory cardiac dysfunction. Cell.

[44] Zhao G., Lu H., Chang Z., Zhao Y., Zhu T., Chang L., Guo Y., Garcia-Barrio M.T., Chen Y.E., Zhang J. (2021). Single-cell RNA sequencing reveals the cellular heterogeneity of aneurysmal infrarenal abdominal aorta. Cardiovasc. Res..

[45] Rizzo G., Gropper J., Piollet M., Vafadarnejad E., Rizakou A., Bandi S.R., Arampatzi P., Krammer T., DiFabion N., Dietrich O. (2023). Dynamics of monocyte-derived macrophage diversity in experimental myocardial infarction. Cardiovasc. Res..

[46] Wang Z., Xie L., Ding G., Song S., Chen L., Li G., Xia M., Han D., Zheng Y., Liu J. (2021). Single-cell RNA sequencing of peripheral blood mononuclear cells from acute Kawasaki disease patients. Nat. Commun..

[47] Luo W.W., Tong Z., Cao P., Wang F.B., Liu Y., Zheng Z.Q., Wang S.Y., Li S., Wang Y.Y. (2022). Transcription-independent regulation of STING activation and innate immune responses by IRF8 in monocytes. Nat. Commun..

[48] Rentsendorj A., Sheyn J., Fuchs D.T., Daley D., Salumbides B.C., Schubloom H.E., Hart N.J., Li S., Hayden E.Y., Teplow D.B. (2018). A novel role for osteopontin in macrophage-mediated amyloid-β clearance in Alzheimer’s models. Brain Behav. Immun..

[49] Capuccini B., Lin J., Talavera-López C., Khan S.M., Sodenkamp J., Spaccapelo R., Langhorne J. (2016). Transcriptomic profiling of microglia reveals signatures of cell activation and immune response, during experimental cerebral malaria. Sci. Rep..

[50] Xu C., Xu H., Dai X., Gui S., Chen J. (2025). Effects and mechanism of combination of Platycodon grandiflorum polysaccharides and Platycodon saponins in the treatment of chronic obstructive pulmonary disease rats through the gut-lung axis. J. Ethnopharmacol..

[51] Zhai R., Xu H., Hu F., Wu J., Kong X., Sun X. (2020). Exendin-4, a GLP-1 receptor agonist regulates retinal capillary tone and restores microvascular patency after ischaemia-reperfusion injury. Br. J. Pharmacol..

[52] Campregher C., Luciani M.G., Gasche C. (2008). Activated neutrophils induce an hMSH2-dependent G2/M checkpoint arrest and replication errors at a (CA)13-repeat in colon epithelial cells. Gut.

[53] Smirnov A., Daily K.P., Gray M.C., Ragland S.A., Werner L.M., Brittany Johnson M., Eby J.C., Hewlett E.L., Taylor R.P., Criss A.K. (2023). Phagocytosis via complement receptor 3 enables microbes to evade killing by neutrophils. J. Leukoc. Biol..

[54] Edmisson J.S., Tian S., Armstrong C.L., Vashishta A., Klaes C.K., Miralda I., Jimenez-Flores E., Le J., Wang Q., Lamont R.J. (2018). Filifactor alocis modulates human neutrophil antimicrobial functional responses. Cell. Microbiol..

[55] Nazari A., Khorramdelazad H., Hassanshahi G., Day A.S., Sardoo A.M., Fard E.T., Abedinzadeh M., Nadimi A.E. (2017). S100A12 in renal and cardiovascular diseases. Life Sci..

[56] Foell D., Kane D., Bresnihan B., Vogl T., Nacken W., Sorg C., Fitzgerald O., Roth J. (2003). Expression of the pro-inflammatory protein S100A12 (EN-RAGE) in rheumatoid and psoriatic arthritis. Rheumatology.

[57] Peng Z., Chen H., Wang M. (2023). Identification of the biological processes, immune cell landscape, and hub genes shared by acute anaphylaxis and ST-segment elevation myocardial infarction. Front. Pharmacol..

[58] Chen H., Lu S., Guan J., Zhu X., Sun F., Huang J., Zhu J., Wang J., Zhen Z., Que Y. (2021). Identification of prognostic immune-related genes in rhabdoid tumor of kidney based on TARGET database analysis. Aging.

[59] Kulohoma B.W., Marriage F., Vasieva O., Mankhambo L., Nguyen K., Molyneux M.E., Molyneux E.M., Day P.J.R., Carrol E.D. (2017). Peripheral blood RNA gene expression in children with pneumococcal meningitis: A prospective case-control study. BMJ Paediatr. Open.

[60] Thaikruea L. (2023). Differences in clinical manifestations between cases stung by single-tentacle and multiple-tentacle box jellyfish over two decades. Heliyon.

[61] de Bruin A.M., Libregts S.F., Valkhof M., Boon L., Touw I.P., Nolte M.A. (2012). IFNγ induces monopoiesis and inhibits neutrophil development during inflammation. Blood.

[62] Xie X., Shi Q., Wu P., Zhang X., Kambara H., Su J., Yu H., Park S.Y., Guo R., Ren Q. (2020). Single-cell transcriptome profiling reveals neutrophil heterogeneity in homeostasis and infection. Nat. Immunol..

